# A systematic review on academic research productivity of postgraduate students in low- and middle-income countries

**DOI:** 10.1186/s12961-018-0360-7

**Published:** 2018-08-28

**Authors:** E. A. Obuku, J. N. Lavis, A. Kinengyere, R. Ssenono, M. Ocan, D. K. Mafigiri, F. Ssengooba, C. Karamagi, N. K. Sewankambo

**Affiliations:** 1 Clinical Epidemiology Unit, Department of Medicine, School of Medicine, College of Health Sciences, Makerere University, PO Box 7072, Kampala, Uganda; 2 McMaster Health Forum, Centre for Health Economics and Policy Analysis, Department of Health Research Methods, Evidence and Impact, and Department of Political Science, McMaster University, Hamilton, Canada; 3 Department of Health Policy and Planning, School of Public Health, College of Health Sciences, Makerere University, Kampala, Uganda; 4 Department of Social Work and Social Administration, College of Humanities and Social Sciences, Makerere University, Kampala, Uganda; 5 Sir Albert Cook Library, College of Health Sciences, Makerere University, Kampala, Uganda; 6 Department of Global Health and Population, Harvard School of Public Health, Harvard University, Cambridge, MA United States of America; 7 Center for Social Science Research on AIDS, Department of Anthropology, College of Arts and Sciences, Case Western Reserve University, Cleveland, OH United States of America; 8 The African Centre for Systematic Reviews and Knowledge Translation, Makerere University, Kampala, Uganda; 9 Faculty of Epidemiology and Population Health, London School of Hygiene and Tropical Medicine, London, United Kingdom

**Keywords:** Student, Productivity, Publication, Citation, Evidence-informed health policy, Knowledge translation

## Abstract

**Background:**

While several individual studies addressing research productivity of post-graduate students are available, a synthesis of effective strategies to increase productivity and the determinants of productivity in low-income countries has not been undertaken. Further, whether or not this research from post-graduate students’ projects was applied in evidence-informed decision-making was unknown. Therefore, we conducted a systematic review of literature to identify and assess the effectiveness of approaches that increase productivity (proportion published) or the application (proportion cited) of post-graduate students’ research, as well as to assess the determinants of post-graduate students’ research productivity and use.

**Methods:**

We conducted a systematic review as per our a priori published protocol, also registered in PROSPERO (CRD42016042819). We searched for published articles in PubMed/MEDLINE and the ERIC databases through to July 2017. We performed duplicate assessments for included primary studies and resolved discrepancies by consensus. Thereafter, we completed a structured narrative synthesis and, for a subset of studies, we performed a meta-analysis of the findings using both fixed and random effects approaches. We aligned our results to the Preferred Reporting Items for Systematic Reviews and Meta-Analyses (PRISMA) statement.

**Results:**

We found 5080 articles in the PubMed (*n* = 3848) and ERIC (*n* = 1232) databases. After excluding duplicates (*n* = 33), we screened 5047 articles, of which 5012 were excluded. We then retrieved 44 full texts and synthesised 14, of which 4 had a high risk of bias. We did not find any studies assessing effectiveness of strategies for increasing publication nor citations of post-graduate research projects. We found an average publication proportion of 7% (95% CI 7–8%, Higgins I-squared 0.0% and Cochran’s Q *p* < 0.01) and 23% (95% CI 17–29%, Higgins I-squared of 98.4% and Cochran’s Q, *p* < 0.01) using fixed effects and random effects models, respectively. Two studies reported on the citation of post-graduate students’ studies, at 17% (95% CI 15–19%) in Uganda and a median citation of 1 study in Turkey (IQR 0.6–2.3). Only one included study reported on the determinants of productivity or use of post-graduate students’ research, suggesting that younger students were more likely to publish and cohort studies were more likely to be published.

**Conclusions:**

We report on the low productivity of post-graduate students’ research in low- and middle-income countries, including the citation of post-graduate students’ research in evidence-informed health policy in low- and middle-income countries. Secondly, we did not find a single study that assessed strategies to increase productivity and use of post-graduate students’ research in evidence-informed health policy, a subject for future research.

**Electronic supplementary material:**

The online version of this article (10.1186/s12961-018-0360-7) contains supplementary material, which is available to authorized users.

## Background

Writing a thesis is a fundamental step for post-graduate studies globally [1]. This process inculcates knowledge and skills for scientific enquiry, critical thinking, systematic problem-solving, and appraisal of scientific and lay claims. This if often followed by dissemination of the thesis results to the scientific community, of which publication in a peer-reviewed journal is the highest and most respected form [2]. However, how much of this thesis work appears only in theses, and its citation particularly in public policy-related work, in low- and middle-income countries is the subject of our systematic review.

Our first aim was to conduct a systematic review of literature that identifies and assesses the effectiveness of approaches that increase productivity (proportion published) or the application (proportion cited) of post-graduate students’ research. Our second aim was to assess the determinants of post-graduate students’ research productivity and use.

## Methods

We registered our protocol a priori in PROSPERO (CRD42016042819) and thereafter published it in a peer-reviewed journal [3]. We thus present an overview of the methodological approach and highlight differences between the protocol and the actual conduct of this systematic review.

### Search strategy

#### Electronic search

We report the electronic search for the PubMed/Medline database only (https://www.ncbi.nlm.nih.gov/pubmed/). We combined terms using Boolean logic ‘OR’ for synonyms and ‘AND’ across elements of PICOS (Population Intervention Comparison Outcome and Study design), as follows: ‘medicine’, ‘nursing’, ‘dentistry’, ‘pharmacy’ and ‘public health’ described the professional fields of interest; while ‘degree’, ‘doctor’, ‘post-doctor’, ‘master’, ‘fellow’, ‘resident’, ‘student’, ‘trainee’, ‘graduate’ and ‘post-graduate’ were intermediate transitional terms for the population of interest, setting or interventions. The terms describing the interventions of interest included were ‘mentor’, ‘grant’, ‘fund’, ‘supervise’, ‘workshop’, ‘seminar’, ‘conference’, ‘manuscript writing’, ‘scientific writing’, ‘academic writing’, ‘scholarly writing’, ‘grants writing’, ‘capacity-building’ and ‘research’.

We defined ‘productivity’ as the proportion of dissertations from which at least one manuscript was published and ‘use’ as the proportion of dissertations cited in peer-reviewed articles, technical reports or policy-related documents. Thus, the search terms for the outcome of interest were ‘abstract’, ‘thesis’, ‘dissertation’, ‘publication’, ‘poster session’, ‘poster presentation’, ‘book chapter’, ‘technical report’, ‘policy brief’, ‘policy dialog’, ‘evidence-informed policy’, ‘evidence-based policy’, ‘evidence-informed health policy’, ‘evidence-based health policy’, ‘decision-making’, ‘policy-making’ and ‘dissemination’.

We restricted these search terms to the title or abstract, and included terms for the outcomes to maximise relevance and efficiency. Further, in order to minimise the risk of an empty review, we did not enter specific terms for the study design as we intended to use all evidence types to describe the available range of interventions. See the full search string in Additional file 1. We found articles in French, Persian and Spanish, and used Google translator (https://translate.google.com/) for English translations during screening and full-text review.

#### Additional searches

In our targeted search, we screened the reference lists of included publications and retrieved full texts of articles likely to be eligible for inclusion. We contacted authors of included articles for any literature that they may be aware of.

### Selection of studies

#### Data management

Using EndNote software version *X7* (Thomson Reuters, 2015) we imported all identified titles, excluded duplicates, and screened and grouped these into relevant eligibility categories as described in our Preferred Reporting Items for Systematic Reviews and Meta-analyses (PRISMA) flow chart [4].

#### Minimising bias in study identification and selection

A second reviewer (RS), an Information Scientist, validated the electronic search in PubMed by performing an independent and duplicate search. Similarly, a third reviewer (MO), screened all full texts excluded by the first reviewer (EAO). We resolved differences by discussion and consensus.

#### Criteria for considering inclusion of studies

We included published studies reporting at least one outcome of interest, and reporting on post-graduate research conducted in a low- and middle-income country.

#### Exclusion criteria for ineligible studies

Our exclusion criteria were studies about research conducted by Bachelor’s degree or undergraduate students’ or established university faculty not identified as post-graduate students, studies conducted in high-income settings or in low-income settings but by students from high-income settings, qualitative designs, non-empirical studies, syntheses, editorials or perspectives, studies published earlier than 1990, or those that were totally irrelevant.

#### Data abstraction

We adapted a data extraction tool for observational studies we had developed for a previous systematic review [5]. We then abstracted administrative, study design and primary outcome data on productivity measured as proportion of publications. We further abstracted our secondary outcomes of use of the research as measured by citations, time to publication, conference abstracts and additional outcomes describing the nature of the post-graduate students’ research (predominant types of research, first authorship status of student).

#### Handling of missing data

We denoted variables that were missing as not reported. We did not employ any statistical methods for handling missing data, neither did we contact authors for additional information.

#### Risk of bias of assessment of included studies

We adapted a tool we used in a published systematic review to assess for the risk of bias in the included studies [5]. We considered the following seven aspects of bias [6, 7]: selection bias due to sampling or proportion of responders or baseline characteristics (and confounding), detection bias due to reliability of measurements used, and bias due to method of data analysis used for overall outcome and reporting biases. We categorised risk of bias as high, moderate or low, guided by the descriptive assessment questions in our tool (Additional file 2).

#### Synthesis of included studies

We employed a structured synthesis in which the units of analysis were findings from a single primary study. First, we described the characteristics of the included primary studies. Using the command ‘metaprop’ in Stata version 14.1 (Stata, College Station, Texas, USA), we constructed forest plots for proportions of published post-graduate work for both fixed and random effects models. The kind of data we abstracted would not permit assessment of the measures of effect, comparing two groups. We thus evaluated single group prevalence of publications, conference abstracts and predominant types of studies of the post-graduate work. We visually explored heterogeneity by inspecting the forest plots and statistically quantifying this using the I-squared statistic and tested for significance using Cochran’s Q. As we found a high level of heterogeneity we conducted a meta-regression testing the variables of duration of study, period of study and geographical region, before conducting a sensitivity analysis by excluding the very large study contributing 93% of the overall combined information. Finally, we wrote a narrative synthesis for the results for which we were unable to perform quantitative meta-analysis.

## Results

### Differences between the published protocol and the actual study

In conducting this review, we employed practical approaches to circumvent unanticipated methodological challenges. We did not contact heads of academic or research departments in target universities as key informants, nor did we search additional grey literature-specific databases due to resource and time constraints of completing this doctoral project. Secondly, we did not find a single article reporting on the effects of strategies to increase productivity or increase the use of post-graduate students research and therefore synthesis was based on other relevant outcomes to map the field for future studies. Third, we did not assess the overall quality of evidence, as we did not find effectiveness studies or studies with comparison groups testing interventions for increasing productivity or use of post-graduate students research [8].

### Systematic review flow, screening and inclusion

**Fig. 1:**
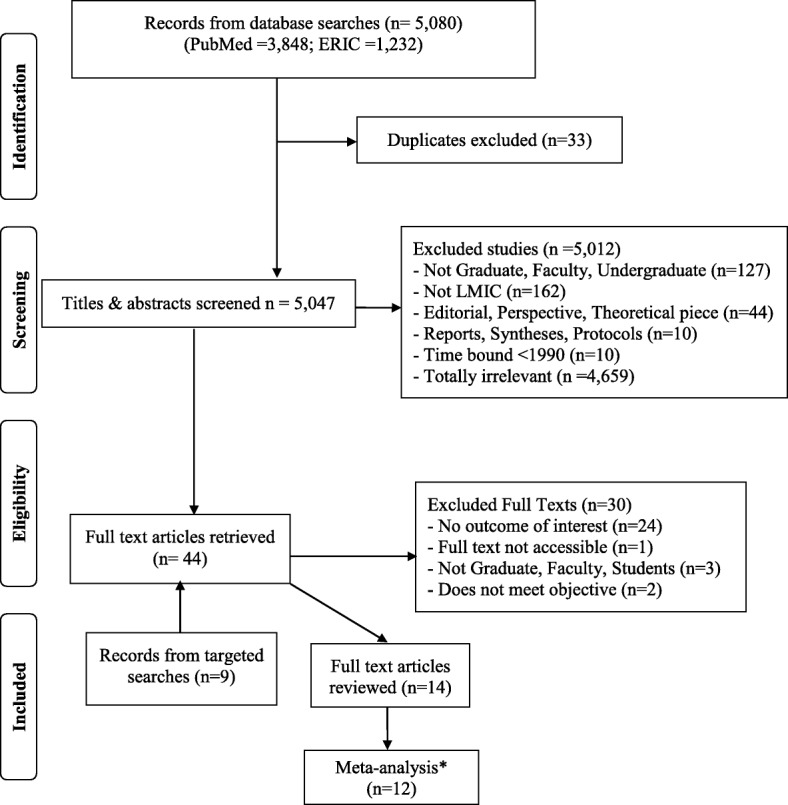
Flow diagram for systematic review on productivity of post-graduate students’ research

Our results are illustrated in the PRISMA flow chart (Fig. 1) and in Table 1, while Table 2 shows a summary of the risk of bias assessments. We retrieved 5080 titles and abstracts from two databases, PubMed (*n* = 3848) and ERIC (*n* = 1232). We updated the search in PubMed only and findings are as recent as July 17, 2017, as it provides the bulk of health-related literature. After excluding 33 duplicates, we screened all 5047 titles or abstracts, excluding 5012 mainly due to irrelevance (*n* = 4659) or various reasons not meeting eligibility criteria (*n* = 353). We retrieved a total of 44 full text articles, of which 9 were from additional targeted searching; we finally reviewed 14 of them, with only 12 in the statistical meta-analysis. The main reason for excluding the full texts was not containing at least one outcome of interest and the two studies were dropped in the meta-analysis either because they lacked the primary outcome [9] or the primary outcome was not reported in a way to permit synthesis [10].

**Fig. 2:**
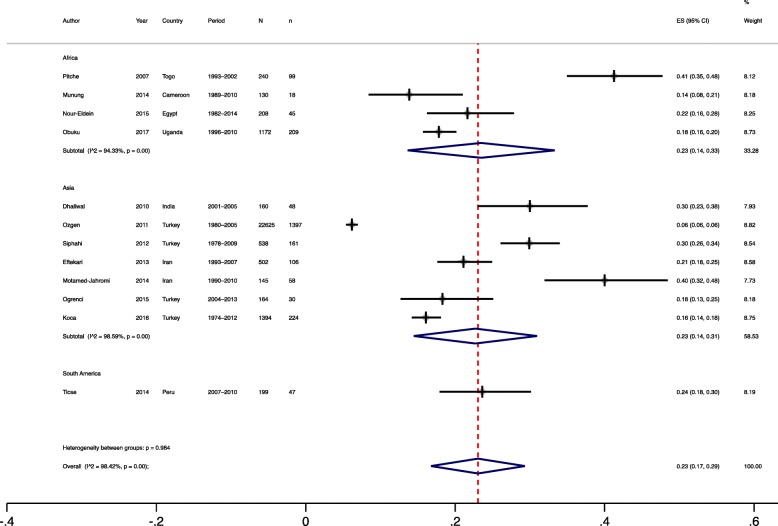
Meta-analysis of productivity of post-graduate students’ research in low- and middle-income countries

**Table 1:** Productivity of post-graduate students’ research in low- and middle-income countries

Region (Author)	Year	Country	Design	Population	Period (years)	^a^Publication *N* (%)	Time to publication (years)	Conference abstract	^b^Citation	Study types		First author
										CSS	RCT	
Afric												
Pitche [12]	2007	Togo	CSS	Masters	1993–2002	240 (41)	–	54%	–	–	–	–
Ahmed [9]	2010	Zambia	CSS	Masters	1986–2009	–	–	–	–	80%	3%	–
Munung [14]	2014	Cameroon	CSS	Masters Doctoral	1989–2010	130 (14)	2.0	11%	–	–	–	23%
Nour-Eldein [15]	2015	Egypt	CSS	Masters	1982–2014	169 (21)	2.0	–	–	77%	6%	62%
				Doctoral		39 (26)						
Obuku [13]	2017	Uganda	CSS	Masters	1996–2010	1172 (18)	2.3	2%	17% (4%)	75%	5%	99%
Asia												
Dhaliwal [2]	2010	India	CSS	Masters	2001–2005	160 (30)	2.81	–	–	44%	23%	54%
Ozgen [11]	2011	Turkey	CSS	Masters	1980–2005	22,625 (6)	–	–	1 (0.6–2.3)	–	–	–
Sipahi [17]	2012	Turkey	CSS	Masters	1978–2009	295 (21)	–	–	–	–	–	80%
				Doctoral		243 (15)						
Eftekari [22]	2013	Iran	CSS	Masters	1993–2007	502 (21)	2.91	–	–	–	–	–
Motamed-Jahromi [16]	2014	Iran	CSS	Masters	1990–2010	145 (40)	3.01	–	–	–	–	–
Ogrenci [23]	2015	Turkey	CSS	Masters	2004–2013	164 (18)	–	–	–	–	–	–
Koca [24]	2016	Turkey	CSS	Masters	1974–2012	1394 (16)	–	–	–	–	–	–
South America												
Linch [10]	2013	Brazil	CSS	Masters Doctoral	2007–2011	90 (103)	–	–	–	–	–	–
Ticse [25]	2014	Peru	CSS	Masters	2007–2010	199 (24)	–	–	–	–	–	–

^a^ Proportion of theses that were published as full text articles in peer-reviewed journals; this is the primary outcome of the systematic review and includes an aggregate of publications in national, regional or international journals

^b^ Proportion of theses that were cited in peer-reviewed journals or policy-related documents; this is the primary outcome of the systematic review and includes an aggregate of publications in national, regional or international journals and in policy-related documents

*CSS* cross sectional studies, *RCT* randomised controlled trials

**Table 2:** Risk of bias assessments for studies on productivity of post-graduate students’ research in low- and middle-income countries

Author	Year	Duration (years)	Selection bias due to sampling	Selection bias due to proportion of responders (> 60%)	Selection bias due to baseline characteristics (and confounding)	Detection bias due to reliability of measurement tools used	Bias due to method of data analysis used for overall outcome	Reporting biases	Overall risk of bias
Africa									
Pitche [12]	2007	1993–2002	Low	Low	Unclear	Low	Low	Low	Low
Munung [14]	2014	1989–2010	High	Unclear	Low	Mod	Low	Low	High
Nour-Eldein [15]	2015	1982–2014	High	Unclear	Mod	Low	Low	Low	High
Obuku [13]	2017	1996–2010	Mod	Low	Low	Mod	Low	Low	Mod
Asia									
Dhaliwal [2]	2010	2001–2005	Low	Low	Unclear	Low	Low	Low	Low
Ozgen [11]	2011	1980–2005	Low	Low	Mod	Low	Low	Low	Low
Sipahi [17]	2012	1978–2009	Low	Unclear	Unclear	Low	Low	Low	Mod
Eftekari [22]	2013	1993–2007	Low	Unclear	Unclear	Low	Low	Low	Mod
Motamed-Jahromi [16]	2014	1990–2010	Low	Unclear	Low	Low	Low	Low	Low
Ogrenci [23]	2015	2004–2013	Mod	Unclear	Unclear	Low	Low	Low	Mod
Koca [24]	2016	1974–2012	High	Unclear	Unclear	High	Low	Mod	High
South America									
Ticse [25]	2014	2007–2010	High	High	Unclear	Low	High	Mod	High

### Description of included studies

The studies we included were from Asia (*n* = 7), Africa (*n* = 5) and South America (*n* = 2), published between 2007 and 2014, including post-graduate students over a 30-year span (1974 to 2014). The smallest study had a sample size of 90, while the largest had 22,625. All studies were about post-graduate students pursuing Masters’ degrees, of which 4 included Doctoral students as well. Importantly, although many studies included cohorts of post-graduate students, all used a cross-sectional analysis. We included 1 study that did not report the primary outcome but described other secondary outcomes, and 7 studies that reported at least an additional outcome of interest.

### Findings on the outcomes of interest

#### Publication proportion of post-graduate students’ research

We included 12 of the 14 review studies in the meta-analysis for the primary outcome, all together contributing work of 27,477 post-graduate students for the meta-analysis (Fig. 2). The proportion of post-graduate students research published ranged from 6% in Turkey [11] to 41% in Togo [12]. We found an average publication proportion of 7% (95% CI 7–8%; Higgins I-squared 0.0% and Cochran’s Q, *p* < 0.01) and 23% (95% CI 17–29%; Higgins I-squared of 98% and Cochran’s Q, *p* < 0.01) using fixed and random effects models, respectively.

#### Meta-regression and sensitivity analyses

After excluding the study by Orzgen et al. [11] as it accounted for 92% of the review sample size, the results were 20% (95% CI 19–21%; Higgins I-squared and Cochran’s Q, *p* < 0.01) by fixed effects and 24% (95% CI 20–29%; Higgins I-squared of 92% and Cochran’s Q, *p* < 0.01) by random effects.

Our meta-regression results were not significant for duration of study span, period of the research or geographical region (data not shown).

#### Citation of post-graduate students’ research

Two studies reported on the citation of studies, which was our surrogate for use of post-graduate students’ research. In Uganda [13], this was 17% overall (95% CI 15–19%) and 4% specific for policy-related documents, while in Turkey [11], the median citation was 1 study (IQR 0.6–2.3).

#### Determinants of post-graduate students’ research

Only one included study reported on the determinants of productivity or use of post-graduate students’ research, and it suggested that younger students were more likely to publish and that cohort studies were more likely to be published [13].

#### Additional outcomes

Six studies reported the time to publication from completion of theses by post-graduate students. The earliest average time to publication was 2 years (Cameroon, Egypt) [14, 15], while the rest were 2.3, 2.8 and 3 years in Uganda [13], India [2] and Iran [16], respectively. Only three studies reported the proportion of abstracts presented in conferences as 54% in Togo, 11% in Cameroon and 2% in Uganda [12–14]. Post-graduate students were first authors of their work in 23%, 54%, 62% and 80% in Cameroon, India, Egypt and Turkey, respectively, and in nearly all their papers in Uganda [2, 12–14, 17]. Cross-sectional studies were the predominant designs of post-graduate students’ research projects (44–80%), while randomised trials were few (3–23%) [2, 9, 13].

## Discussion

### Principal findings

We report that the majority of post-graduate students in low-income countries infrequently publish their research theses. Secondly, the most published studies are cross-sectional in design with hardly any clinical trials, likely because of feasibility considerations with higher logistic demands, particularly for students. Third, it is apparent that post-graduate students are not the first authors in a significant proportion of their published work. Nevertheless, the evidence of citations suggests that post-graduate students’ research work is used in some form.

### Findings in relation to other systematic reviews

Although we did not identify data to support our primary objective, we found a systematic review about interventions for increasing scholarly productivity among residents in the United States and Canada, which are high-income settings [18]. This review mapped the following approaches that were associated with increased scholarly productivity: protected research time, research curricula, research directors, dedicated research days, and research tracks, but with mixed effects on resident presentations or publications. It would be important to extend these single studies by explicitly testing the approaches found to be associated with productivity in low-income countries such as Uganda.

The proportion of publications in our review was relatively low. In a seminal paper by Dickersin et al. [19], students in John Hopkins University in the 1980s were found to publish less than the faculty. Although it is not clear why students published less, we found that students were not necessarily first authors of their work even when they published. Indeed, authorship would be an incentive for career advancement for which students and faculty would benefit. It is possible that power imbalances may explain this finding and, in some instances, discourage would be student authors from publishing.

With only a single study appropriately documenting the determinants of productivity and citation, we could hardly draw firm conclusions. The results herein suggested that younger students were more likely to publish and that cohort studies were more likely to be published. Bullen and Reeve [20] documented that Master of Public Health students in New Zealand mentioned that barriers to publication included a protracted publication process and a negative perception of the importance of the results. Future qualitative investigations in this setting would be informative.

### Systematic review strengths and limitations

This is the first systematic review documenting the academic research productivity of postgraduate students in low- and middle-income countries. We have employed a robust and internationally agreed methodology [21] to conduct this systematic review with a sizeable sample of included studies and students.

Nonetheless, we report two main methodological limitations in our review. First, we did not find a single study assessing the effects of interventions to increase academic research productivity of post-graduate students. This could be explained by the identification of all the relevant studies or university reports, limited by grey literature beyond the reach of our review team. Nonetheless, we employed a robust and comprehensive search strategy beyond the electronic search, which enabled us access to articles for over a 40-year span and in languages other than English. Secondly, identifying the outcome of ‘use’ of students’ research in the policy process or decision-making in health remains a challenge, with citation being an imperfect proxy. An additional limitation was the fact that we did not explore grey literature more effectively, largely due to resources constraints.

### Implications for future research and policy

It is clear that there is a sheer lack of evidence to effectively assess interventions to increase productivity and use of research conducted by post-graduate students in low- and middle-income countries. The cohort studies that exist did not analyse the data in a manner that permits comparisons of groups exposed to specific approaches, an area that can be strengthened. Future studies should be prospective, employing mixed methods to investigate interventions to increase productivity and citation of post-graduate students’ research as well as to identify unique aspects such as publishing in predatory journals. Additionally, it is becoming more common for early career researchers globally to publish during their candidature (PhD by publication), an area that can be examined further. Policy-makers and actors should invest in supporting research in this area.

## Conclusions

We report a low productivity of post-graduate students’ research in low- and middle-income countries, including the use of post-graduate students’ research in evidence-informed health policy. Secondly, we did not find a single study that assessed strategies to increase productivity and use of post-graduate students’ research in evidence-informed health policy, a subject for future research.

## Additional files

Additional file 1: **Supplement 1.** Feasibility of yield of literature of pilot electronic search strategy for post-graduate students’ research. **Supplement 2.** Updated search strategy as at 17th July 2017 in PubMed (https://www.ncbi.nlm.nih.gov/pubmed/). (DOCX 21 kb)

Additional file 2: Risk of bias assessment tool: productivity of post-graduate students’ research in low- and middle-income countries. (DOC 59 kb)
