# Supplementary material for: A systematic review on academic research productivity of postgraduate students in low- and middle-income countries
Source: Health Res Policy Syst. 2018 Aug 28;16:86. doi: 10.1186/s12961-018-0360-7 (PMC6114801; doi:10.1186/s12961-018-0360-7)
Supplement: Supplementary file 1 — Supplement 1. Feasibility of yield of literature of pilot electronic search strategy for post-graduate students’ research. Supplement 2. Updated search strategy as at 17th July 2017 in PubMed (https://www.ncbi.nlm.nih.gov/pubmed/). (DOCX 21 kb) [file 12961_2018_360_MOESM1_ESM.docx]

**Additional file 1**

**Supplement 1: Feasibility of yield of literature of pilot electronic search strategy for post-graduate student’s research**

| **Search number**  **(Data base)** | **Search terms (and date)** | **Number of hits***  **(Relevant)** |
| --- | --- | --- |
| #7  (PubMed) | *(((((((Medicine[tiab] OR Nursing[tiab] OR Dentistry[tiab] OR Pharmacy[tiab] OR “Public health”))) AND ((Degree*[tiab] OR Doctor*[tiab] OR Post-doc*[tiab] OR PhD[tiab] OR Master*[tiab] OR Fellow*[tiab] OR Residen*[tiab] OR Student*[tiab] OR Trainee*[tiab] OR Graduate*[tiab] OR Post-grad*[tiab])))) AND ((Mentor*[tiab] OR Grantee [tiab] OR Fund*[tiab] OR Supervis*[tiab] OR Workshop*[tiab] OR Seminar*[tiab] OR Conference*[tiab] OR “Manuscript-writing”[tiab] OR "Scientific-writing”[tiab] OR "Academic-writing”[tiab] OR "Scholarly-writing”[tiab] OR “Grants-writing”[tiab] OR “Capacity building”[tiab] OR Research[tiab])))) AND ((Abstract*[tiab] OR Thesis[tiab] OR Theses[tiab] OR Dissertation*[tiab] OR publication*[tiab] OR “Poster session” OR “Poster presentation” [tiab] OR “Book chapter” [tiab] OR “Technical report”[tiab] OR “Policy brief”[tiab] OR “Policy dialogue” [tiab] OR “Evidence informed policy”[tiab] OR “Evidence based policy”[tiab] OR “Evidence informed health policy”[tiab] OR “Evidence based health policy”[tiab] OR “Decision making”[tiab] OR “Policy making”[tiab] OR Dissemination[tiab]))* | *3,460 (*^§^*176, 5 %) |

**Number of article titles and abstracts as at 21.06.2016*

*^§^Sorted by relevance and initial screening of titles and abstracts*

**Supplement 2: Updated search strategy as at 17^th^ July 2017 in PubMed** (<https://www.ncbi.nlm.nih.gov/pubmed>)

**Terms describing the population/field of interest**

#1

Medicine[tiab] OR Nursing[tiab] OR Dentistry[tiab] OR Pharmacy[tiab] OR “Public health”

**Intermediate terms, which describe the population of interest or setting or interventions**

#2

Degree*[tiab] OR Doctor*[tiab] OR Post-doc*[tiab] OR PhD[tiab] OR Master*[tiab] OR Fellow*[tiab] OR Residen*[tiab] OR Student*[tiab] OR Trainee*[tiab] OR Graduate*[tiab] OR Post-grad*[tiab]

**Terms describing the interventions of interest**

#3

Mentor*[tiab] OR Grantee [tiab] OR Fund*[tiab] OR Supervis*[tiab] OR Workshop*[tiab] OR Seminar*[tiab] OR Conference*[tiab] OR “Manuscript-writing”[tiab] OR "Scientific-writing”[tiab] OR "Academic-writing”[tiab] OR "Scholarly-writing”[tiab] OR “Grants-writing”[tiab] OR “Capacity building”[tiab] OR Research[tiab]

**Terms describing the outcomes of interest**

#4

Abstract*[tiab] OR Thesis[tiab] OR Theses[tiab] OR Dissertation*[tiab] OR publication*[tiab] OR “Poster session” OR “Poster presentation” [tiab] OR “Book chapter” [tiab] OR “Technical report”[tiab] OR “Policy brief”[tiab] OR “Policy dialogue” [tiab] OR “Evidence informed policy”[tiab] OR “Evidence based policy”[tiab] OR “Evidence informed health policy”[tiab] OR “Evidence based health policy”[tiab] OR “Decision making”[tiab] OR “Policy making”[tiab] OR Dissemination[tiab]

#5= (#1 AND #2)

(((Medicine[tiab] OR Nursing[tiab] OR Dentistry[tiab] OR Pharmacy[tiab] OR “Public health”))) AND ((Degree*[tiab] OR Doctor*[tiab] OR Post-doc*[tiab] OR PhD[tiab] OR Master*[tiab] OR Fellow*[tiab] OR Residen*[tiab] OR Student*[tiab] OR Trainee*[tiab] OR Graduate*[tiab] OR Post-grad*[tiab]))

#6= (#5 AND #3)

(((((Medicine[tiab] OR Nursing[tiab] OR Dentistry[tiab] OR Pharmacy[tiab] OR “Public health”))) AND ((Degree*[tiab] OR Doctor*[tiab] OR Post-doc*[tiab] OR PhD[tiab] OR Master*[tiab] OR Fellow*[tiab] OR Residen*[tiab] OR Student*[tiab] OR Trainee*[tiab] OR Graduate*[tiab] OR Post-grad*[tiab])))) AND ((Mentor*[tiab] OR Grantee [tiab] OR Fund*[tiab] OR Supervis*[tiab] OR Workshop*[tiab] OR Seminar*[tiab] OR Conference*[tiab] OR “Manuscript-writing”[tiab] OR "Scientific-writing”[tiab] OR "Academic-writing”[tiab] OR "Scholarly-writing”[tiab] OR “Grants-writing”[tiab] OR “Capacity building”[tiab] OR Research[tiab]))

#7= (#6+#4)

(((((((Medicine[tiab] OR Nursing[tiab] OR Dentistry[tiab] OR Pharmacy[tiab] OR “Public health”))) AND ((Degree*[tiab] OR Doctor*[tiab] OR Post-doc*[tiab] OR PhD[tiab] OR Master*[tiab] OR Fellow*[tiab] OR Residen*[tiab] OR Student*[tiab] OR Trainee*[tiab] OR Graduate*[tiab] OR Post-grad*[tiab])))) AND ((Mentor*[tiab] OR Grantee [tiab] OR Fund*[tiab] OR Supervis*[tiab] OR Workshop*[tiab] OR Seminar*[tiab] OR Conference*[tiab] OR “Manuscript-writing”[tiab] OR "Scientific-writing”[tiab] OR "Academic-writing”[tiab] OR "Scholarly-writing”[tiab] OR “Grants-writing”[tiab] OR “Capacity building”[tiab] OR Research[tiab])))) AND ((Abstract*[tiab] OR Thesis[tiab] OR Theses[tiab] OR Dissertation*[tiab] OR publication*[tiab] OR “Poster session” OR “Poster presentation” [tiab] OR “Book chapter” [tiab] OR “Technical report”[tiab] OR “Policy brief”[tiab] OR “Policy dialogue” [tiab] OR “Evidence informed policy”[tiab] OR “Evidence based policy”[tiab] OR “Evidence informed health policy”[tiab] OR “Evidence based health policy”[tiab] OR “Decision making”[tiab] OR “Policy making”[tiab] OR Dissemination[tiab]))
