# Supplementary material for: A systematic review on academic research productivity of postgraduate students in low- and middle-income countries
Source: Health Res Policy Syst. 2018 Aug 28;16:86. doi: 10.1186/s12961-018-0360-7 (PMC6114801; doi:10.1186/s12961-018-0360-7)
Supplement: Supplementary file 2 — Risk of bias assessment tool: productivity of post-graduate students’ research in low- and middle-income countries. (DOC 59 kb) [file 12961_2018_360_MOESM2_ESM.doc]

**Additional file 2: Risk of bias assessment tool: Productivity of post–graduate students’ research in low- and middle-income countries**

| **S/n** | **Type of bias** | **Signaling question** | **Response to the evaluation** | | | |
| --- | --- | --- | --- | --- | --- | --- |
|  |  |  | **Yes** | **No** | **Can’t tell** | **Result and comment (LR/HR/NC)** |
| **01** | **Selection bias due to sampling** | Were the sampling of post–graduates representative or were full registers of all post–graduates used? Examples of random sampling techniques include simple random, multi–stage, stratified and cluster sampling. Non–random methods are purposive, convenience, alternate sequencing; et cetera. |  |  |  |  |
| **02** | **Selection bias due to proportion of responders** | What was the response rate obtained? Did this response rate influence the measurement of the main out–come of publication output? (Hint: >59.9% is the cut–off) |  |  |  |  |
| **03** | **Selection bias due to baseline characteristics (& confounding)** | Was the sample (post–graduate students) selected such that the baseline characteristics influenced the outcome of interest (publication output)? For example excluding students who were less likely to publish e.g. female married students or only those who completed their theses in time? |  |  |  |  |
| **04** | **Detection bias due to reliability of measurements used** | Was the process of searching for published studies clear and comprehensive? Were more than two databases searched? Were local and international databases searched? Were there additional search strategies? (Contacting authors; supervisors; conducting hand searches; national journals; conference proceedings) |  |  |  |  |
| **05** | **Bias due to method of data analysis used for overall outcome** | Was the method used to analyze the data appropriate to the study design and to the outcome of interest? Helpful is a clear description of analysis method used for example “intention–to–treat”, “as–treated”, “complete–case” analyses or consideration given to sampling techniques such as clustering effects or stratification; and if any exclusions were explained or justified. Noteworthy is the denominator used relative to the response rate or missing data; is the “missingness” informative or not? |  |  |  |  |
| **06** | **Reporting biases** | Any selective reporting biases identified in the way outcomes are reported? |  |  |  |  |
| **07** | **Overall Result** | Record your overall impression as follows  High: >2 HR  Moderate: >2 MR  Low: No HR, <2 MR |  | | |  |

**LR: Low risk; MR: Moderate risk; HR: High risk; NC: Unclear**
